# Comparative transcriptome analysis identifies candidate genes related to sucrose accumulation in longan (*Dimocarpus longan* Lour.) pulp

**DOI:** 10.3389/fpls.2024.1379750

**Published:** 2024-04-05

**Authors:** Yun Li, Rui Ren, Ruoyun Pan, Yuying Bao, Tao Xie, Lihui Zeng, Ting Fang

**Affiliations:** College of Horticulture, Institute of Genetics and Breeding in Horticultural Plants, Fujian Agriculture and Forestry University, Fuzhou, China

**Keywords:** longan, sucrose, sucrose-phosphate synthase, interact network, transcription factor

## Abstract

Sucrose content is one of the important factors to determine longan fruit flavor quality. To gain deep insight of molecular mechanism on sucrose accumulation in longan, we conducted comparative transcriptomic analysis between low sucrose content longan cultivar ‘Qingkebaoyuan’ and high sucrose content cultivar ‘Songfengben’. A total of 12,350 unique differentially expressed genes (DEGs) were detected across various development stages and different varieties, including hexokinase (HK) and sucrose-phosphate synthase (SPS), which are intricately linked to soluble sugar accumulation and metabolism. Weighted gene co-expression network analysis (WGCNA) identified magenta module, including *DlSPS* gene, was significantly positively correlated with sucrose content. Furthermore, transient expression unveiled *DlSPS* gene play crucial role in sucrose accumulation. Moreover, 5 transcription factors (MYB, ERF, bHLH, C2H2, and NAC) were potentially involved in *DlSPS* regulation. Our findings provide clues for sucrose metabolism, and lay the foundation for longan breeding in the future.

## 1 Introduction

Longan (*Dimocarpus longan* Lour.) is a typical tropical and subtropical fruit tree which from the Sapindaceae family (Wang et al., 2022). It is widely cultivated in numerous countries, including China, Australia, East Malaysia, and Sri Lanka. Notably, China boasts the largest planting area and highest yield of longan (Lithanatudom et al., 2017). This fruit is rich in nutritional and medical values, earning the nickname of a “treasure among fruits” (Huang et al., 2021). With the enhancement of people’s living standards, evolving consumption patterns, and the globalization of the product market, consumers are increasingly discerning about fruit flavors. Sugar content plays a significant role in determining fruit flavor and quality.

During photosynthesis, sugars are key products of carbon and energy utilization. Within plants, they not only serve as a carbon source but also function as signaling molecules that respond to environmental signals, developmental cues, and metabolic information (Lastdrager et al., 2014). Furthermore, sweetness, an essential component of fruit organoleptic quality, has a direct impact on consumers’ preferences for fresh fruit (Fang et al., 2020a; Fang et al., 2020b). The intensity of sweetness depends on the sugar composition and content as different types of soluble sugars possess varying degrees of sweetness relative to one another (Ma et al., 2015).

In plant, sugars are synthesized in leaves (the source) and transported to sink cells using sugar transporters or plasmodesmata from sieve element-companion cell (SE-CC). These sugars are then stored in vacuoles through the action of sugar transporters located on the vacuole membrane (Chen et al., 2015; Chen et al., 2021; Zhu et al., 2021). Notably, sucrose serves as the primary carbohydrate shuttled from photosynthetically active source tissues to heterotrophic sink tissues, playing a pivotal role in the resource allocation system (Ruan, 2014; Hedrich et al., 2015; Chen, 2022). The accumulation of sucrose in fruits is determined by its transportation and metabolism. Multiple key enzymes were involved in sucrose metabolism, including sucrose phosphate synthase (SPS), sucrose synthase (SS), and invertase (INV) (Ren and Zhang, 2013). SPS converts uridine diphosphate-glucose (UDPG) and fructose-6 phosphate (F6P) into sucrose (Ma et al., 2020). Overexpression of a SPS gene from *Arabidopsis* family in tobacco resulted in significantly higher sucrose concentrations in the storage sinks compared to WT plants (Park et al., 2008). The loss of the two SPS genes in *Arabidopsis thaliana* confined sucrose synthesis (Volkert et al., 2014). SuSy, which catalyzes reversible reactions between sucrose and UDPG, participated in the sucrose degradation pathway (Stein and Granot, 2019). A single nucleotide polymorphism (SNP) variation in the *SoSUS* gene has been found to be significantly associated with sugar-related traits (Khanbo et al., 2023). Overexpression of *PtSS3* alters fructose, glucose, and sucrose levels in *Populus tomentosa* stems and leaves (Li et al., 2020). INV, including acid invertases (AINV) and neutral invertases (NINV), catalyzes sucrose into fructose and glucose (Chen et al., 2021). Inhibition of *NtNINV10* gene expression decreased glucose and fructose levels in tobacco leaves (Cheng et al., 2023). Additionally, hexokinase (HK) and fructokinase (FK) play important roles in glucose metabolism. Overexpression of *Prunus HXK3* in *Arabidopsis* significantly decreased soluble sugars content compared to wild-type plants (Pérez-Díaz et al., 2021). Fruits from transgenic apple trees that overexpressed *MdFRK2* exhibited significantly elevated levels of fructose compared to wild-type (Su et al., 2023).

Transcription factors (TFs) play important roles in fruit sugar accumulation by influencing the expression levels of sugar metabolism and transport genes (Li et al., 2020; Yue et al., 2020; Wang et al., 2021; Gao et al., 2024; Zhang et al., 2024). In citrus, *CitZAT5* adjusts the hexose proportion by regulating the expression of two sugar transport genes (Fang et al., 2023). In apples, *MdAREB1.1/1.2* regulate sugar concentration by binding to the promoter of *MdTST1/2*, thereby altering its expression (Zhu et al., 2023). In pears, *PuPRE6* and *PuMYB12* act as antagonistic complexes to regulate the transcription of *PuSUT4-like* and sucrose accumulation (Gao et al., 2024), while *PbrbZIP15* promotes soluble sugar accumulation by activating *PbrXylA1* transcription during development (Jia et al., 2023).

To investigate the potential molecular regulation mechanism of sucrose metabolism and accumulation in longan, a comparative transcriptomic analysis was performed in high sucrose content cultivar ‘Songfengben’ and low sucrose content cultivar ‘Qingkebaoyuan’ during fruit development. Combined DEG and WGCNA analysis, several key genes involved in sugar accumulation were uncovered, such as sucrose phosphate synthase (*DlSPS*) and hexokinase (*DlHK*). Transient overexpressing the *DlSPS* gene in strawberry fruit increased sucrose accumulation. The findings shed light on the regulatory networks involved in sucrose accumulation during fruit development in longan, which will provide a scientific basis for breeding for this trait.

## 2 Materials and methods

### 2.1 Plant materials and measurement of fruit sugar content

Two longan cultivars with contrasting sucrose contents, namely, ‘Songfengben’ (high sucrose accumulating type, SFB) and ‘Qingkebaoyuan’ (low sucrose accumulating type, QKBY), were used in this study for RNA-seq. The cultivars were collected at Fruit Research Institute, Fujian Academy of Agricultural Sciences, Fuzhou, China. Fruits of uniform size and free from visible defects were collected from three trees, with ten fruits from one tree were regarded as one replication, at 60, 90, and 120 days after flowering (DAF), respectively. After removing the peel and seed coat, the pulp was immediately preserved in liquid nitrogen and stored at −80°C until further analysis. The ‘Hongyan’ strawberry cultivar used for transient transformation were grown in a greenhouse with 16 h/8 h light conditions at 22°C.

Sugar content was measured using high performance liquid chromatography (HPLC) according to our reported protocol (Fang et al., 2020a). In brief, 0.5 g of fruit sample powder was dissolved in 6 mL deionized water and ultrasonic treatment for 15 min. After centrifugation at 5,000 rpm for 15 min at 4°C, the supernatant was collected and then filtered through a 0.22 μm Sep-Pak filter (ANPEL, Shanghai, China). Sugar content was detected using a 1260 HPLC system (Agilent, USA) with refractive index detector (RID). The mobile phase was deionized water and elution flow rate was 0.5 mL/min.

### 2.2 RNA extraction and RNA-seq analysis

The RNAprep Pure PlantPlus Kit (Tiangen, Beijing, China) was used to extract RNA in accordance with the manufacturer’s instructions. Subsequently, the quality of the extracted RNA was assessed using both a NanoDrop 2000 spectrophotometer (Thermo, USA) and an Agilent 2100 Bioanalyzer (Agilent, USA). For library preparation, 1 μg of RNA from each sample was used as the starting material. The NEBNext Ultra™ RNA Library Prep Kit for Illumina (NEB, USA) was then utilized to construct the sequencing librariesaccording to the manufacturer’s guidelines. Sequencing was carried out on the Illumina NovaSeq 6000 platform, yielding paired-end RNA-seq reads. Raw data underwent preprocessing to eliminate adapter sequences, reads with poly-N sequences, and those of poor quality. The clean reads were then aligned to the ‘Honghezi’ longan genome (Lin et al., 2017) using HISAT2 with standard settings (Kim et al., 2015). The expression levels of genes were calculated using fragments per kilobase of transcript per millionmapped reads (FPKM). The differentially expressed genes (DEGs) analysis was performed using the DEseq2 R package, with an absolute log2 (fold change) ≥1 and false discovery rate ≤ 0.05 (Love et al., 2014). Both Gene Ontology (GO) and Kyoto Encyclopedia of Genes and Genomes (KEGG) pathway analyses were facilitated by BMKcloud (https://www.biocloud.net/).

### 2.3 Weighted gene co-expression network analysis

The WGCNA was conducted using the WGCNA plug-in (https://github.com/ShawnWx2019/WGCNA-shinyApp). The modules were constructed using the automatic network build function with the following parameters: power=22 and minModuleSize=30. Module-trait associations were considered statistically significant at p<0.05. Genes that were significantly associated with traits were identified within the modules, and their relationships were analyzed. The co-expression network was mapped using Cytoscape V3.8.2 software.

### 2.4 Quantitative real-time PCR

The qRT-PCR analysis were performed on the LightCycler 96 Real-Time PCR Systems (Roche, USA) with the TB Green Premix Ex Taq II (supplied by Takara in Dalian, China) and the primer sequences listed in **Supplementary Table S1**. *Actin* was chosen as the reference gene (Jue et al., 2018). The relative gene expression levels were calculated using the 2^-ΔΔCt^ method (Livak and Schmittgen, 2001).

### 2.5 Transient transformation in strawberry

The coding region of *DlSPS* gene was amplified from the cDNA of ‘SFB’ fruit and inserted into the site of *pSAK277* vector between Hind III and XbaI enzyme sites using ClonExpress^®^ IIOne Step Cloning Kit (Vazyme, China). Primer sequences are listed in **Supplementary Table S1**. The recombinant constructs *pSAK277*-*DlSPS* and the empty vector *pSAK277* were individually transferred into *Agrobacterium tumefaciens* EHA105 with the freeze-thaw method. Transient overexpressed in strawberry fruits was conducted as previously described (Pi et al., 2019).

### 2.6 Statistical analysis

Values are means ± standard deviation (SD) of three biological replicates. The significant differences between mean values was determined by the Student’s t test (*p<0.05 and **p<0.01) calculated using SPSS 21.0 (IBM, USA).

## 3 Results

### 3.1 Changes in soluble sugars content during the developing of ‘QKBY’ and ‘SFB’ longon fruits

**Figure 1:**
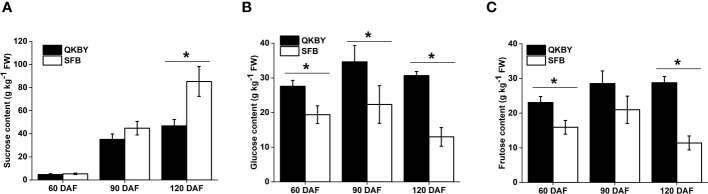
Changes in sugar contents in two longan cultivars during fruit development. **(A)** Sucrose; **(B)** Glucose; **(C)** Fructose. Small star above the bars indicate significant differences (* p ≤ 0.05).

The soluble sugar component and content in two cultivars was mainly carried out at 60, 90, and 120 DAF. Throughout the developmental process, the sucrose content increased most rapidly and becomes the dominant soluble sugar at mature stage (**Figure 1**). Notably, ‘SFB’ fruit exhibits a significantly higher sucrose content compared to ‘QKBY’ fruit at 120 DAF. However, no significant differences are observed at other developmental stages (**Figure 1A**). The glucose and fructose contents showed a slight increase from 60 DAF to 90 DAF, followed by a slight decrease from 90 DAF to 120 DAF. Differences in glucose content between the two cultivars are significant at three stages, while significant differences in fructose content are observed at 60 DAF and 120 DAF (**Figures 1B, C**). Based on the results, we infer that the higher sugar content and sweeter taste of ‘SFB’ longan fruit can be attributed to the higher accumulation of sucrose.

### 3.2 An overview of transcriptome data

To study the transcriptional regulation of soluble sugar accumulation in longan, RNA-seq was conducted on two cultivars, ‘SFB’ and ‘QKBY’, during three critical developmental stages. An overview of the sequencing quality is provided in **Supplementary Table S2**. After removing low-quality reads, the number of clean reads per library ranged from 20,418,767 to 81,405,449, with 72.57%-92.28% of the clean reads successfully mapped to the longan genome. The percentage of Q30 bases ranged from 94.60% to 95.63%, indicating the sequencing data was high quality. The GC content of the clean reads varied between 44.19% and 46.14%.

In total, 33,655 genes, including 4,960 novel genes, were identified across all fruit samples tested. For the annotation of these novel unigenes, we conducted a comprehensive search in public databases, including NR, Swiss-Prot, eggNOG, COG, GO, Pfam, KOG, and KEGG. The majority of the novel unigenes were annotated in the NR protein database (2539), followed by eggNOG (2204), Swiss-Prot (1465), KOG (1448), GO (1369), Pfam (1216), KEGG (669), and COG (363) (**Supplementary Table S3**).

**Figure 2:**
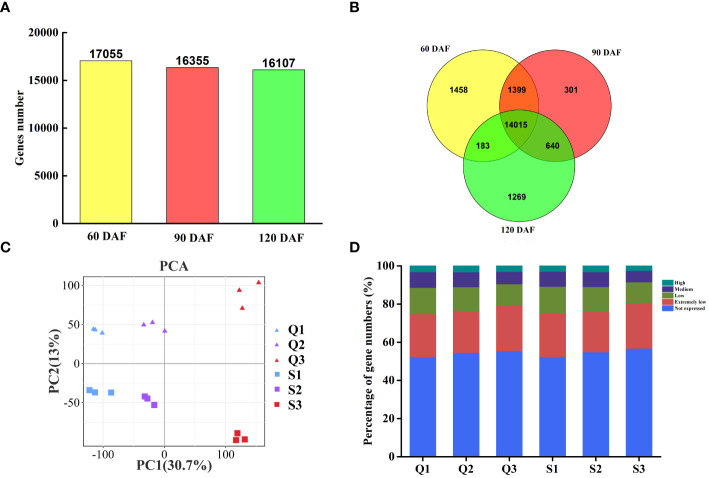
Gene expression and correlation between the transcriptomes of three representative stages each of two longan cultivars. **(A)** The proportion of expressed genes in two longan cultivars of three typical stages. **(B)** Venn diagram of expressed genes in two longan cultivars of three typical stages. **(C)** Principal component analysis (PCA) plot showing clustering of transcriptomes of three representative stages in ‘Songfengben’ and ‘Qingkebaoyuan’. **(D)** Percentage of gene numbers in different categories according to their expression levels in each sample based on the FPKM values. Q1: QKBY at 60 DAF; Q2: QKBY at 90 DAF; Q3: QKBY at 120 DAF; S1: SFB at 60 DAF; S2: SFB at 90 DAF; S3: SFB at 120 DAF.

The gene expression levels among different fruit samples were assessed base on the FPKM value. Genes with FPKM values≥1.0 were considered expressed genes. Among the 33,655 identified genes, 17,055, 16,355, and 16,107 genes were expressed at 60 DAF, 90 DAF, and 120 DAF, respectively (**Figure 2A**). Of these genes, 14,015 were expressed throughout fruit development, while 1,458, 301, and 1,269 were specifically expressed at 60 DAF, 90 DAF, and 120 DAF, respectively (**Figure 2B**). A principal component analysis (PCA) of the expressed genes clustered them into six groups (**Figure 2C**). We further classified the expressed genes into five different groups based on their expression levels: extremely low-expressed genes with FPKM values less than 10, low-expressed genes with 10-30 FPKM, medium-expressed genes with 30-100 FPKM, and highly expressed genes with FPKM values above 100 (**Figure 2D**). Upon comparing the qRT-PCR evaluation of ten genes against the transcriptomic FPKM datasets, the expression patterns were similar to the RNA-Seq data (**Supplementary Figure S1**).

### 3.3 Analyses of DEGs

**Figure 3:**
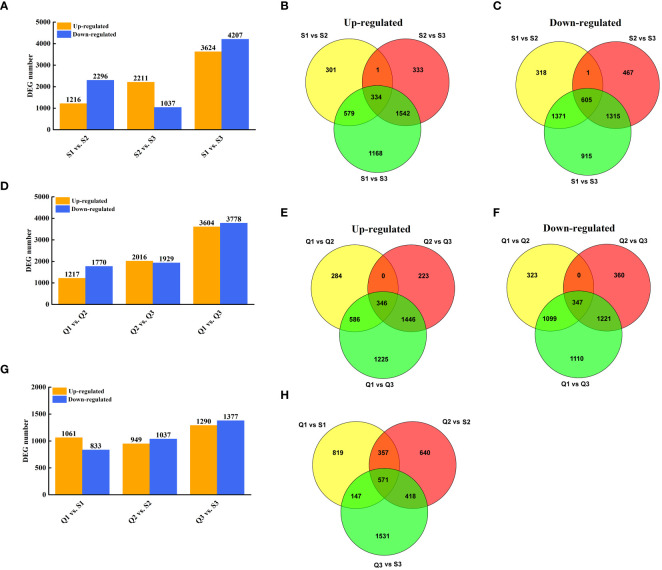
Identification of DEGs at three representative stages each of two longan cultivars. **(A)** The number of DEGs between two stages in ‘SFB’. **(B)** Venn diagram of the number of upregulated DEGs during fruit development in ‘SFB’. **(C)** Venn diagram of the number of downregulated DEGs during fruit development in ‘SFB’. **(D)** The number of DEGs between two stages in ‘QKBY’. **(E)** Venn diagram of the number of upregulated DEGs during fruit development in ‘QKBY’. **(F)** Venn diagram of the number of downregulated DEGs during fruit development in ‘QKBY’. **(G)** The number of DEGs between two cultivars at same stage. **(H)** Venn diagram of the number of DEGs between two cultivars at same stage. Q1: QKBY at 60 DAF; Q2: QKBY at 90 DAF; Q3: QKBY at 120 DAF; S1: SFB at 60 DAF; S2: SFB at 90 DAF; S3: SFB at 120 DAF.

To explore the expression-profile differences between two cultivars, the DEGs analysis was conducted. In ‘SFB’, 7,831 and 3,248 genes were found to be differentially expressed in S3 compared to S1 and S2 (**Figure 3A**). Of these, 3,512 genes were differentially expressed in S2 compared to S1 (**Figure 3A**). Specifically, 334 genes were up-regulated and 605 genes were down-regulated in S3/S1, S3/S2, and S2/S1 (**Figures 3B, C**). Similarly, in ‘QKBY’, 7,382 and 3,945 genes were differentially expressed in Q3 compared to Q1 and Q2 (**Figure 3D**). Among these, 2,987 genes were differentially expressed in Q2 compared to Q1 (**Figure 3D**). Notably, 346 genes were up-regulated and 347 genes were down-regulated in Q3/Q1, Q3/Q2, and Q2/Q1 (**Figures 3E, F**). When comparing ‘QKBY’ and ‘SFB’ at the same fruit development time points, 1,894 (Q1 vs. S1), 1,986 (Q2 vs. S2), and 2,667 (Q3 vs. S3) differentially expressed genes (DEGs) were identified (**Figure 3G**). After excluding redundant genes, a total of 12,350 unique DEGs were detected across various development stages and different longan varieties.

**Figure 4:**
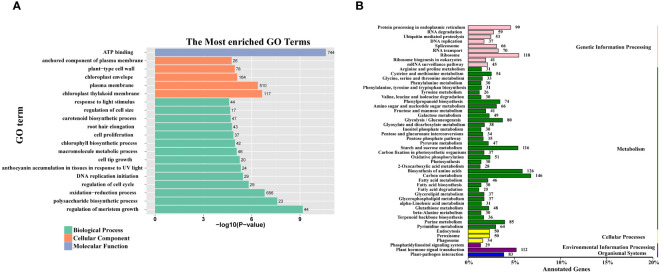
GO enrichment and KEGG class of all DEGs. **(A)** The 20 most significantly GO terms of DEGs. The numerals beside the histogram indicate the number of DEGs. **(B)** The KEGG class of DEGs. The numerals beside the histogram indicate the number of DEGs involved in the pathway.

To gain insight into the functional properties of the DEGs, a comprehensive GO and KEGG analysis was conducted. The DEGs were categorized into three broad domains: molecular functions, cellular components, and biological processes. Within the cellular component category, DEGs were highly enriched in specific subcategories including the anchored component of the plasma membrane, the plant-type cell wall, the chloroplast envelope, the plasma membrane, and the chloroplast thylakoid membrane. In the biological process category, DEGs were further subclassified into a range of processes including response to light stimulus, regulation of cell size, cell proliferation, chlorophyll biosynthesis process, and polysaccharide biosynthetic process. Notably, within the molecular function category, the sole enriched function was ATP binding (**Figure 4A**).

The KEGG pathway classification analysis revealed that the DEGs were significantly associated with several key metabolic pathways. These included fructose and mannose metabolism, starch and sucrose metabolism, glucosinolate biosynthesis, and glycolysis/gluconeogenesis (**Figure 4B**).

### 3.4 Identification of DEGs in soluble sugar accumulation

As shown in **Figure 1**, the sucrose content in ‘SFB’ fruit only exhibit significantly higher than that in ‘QKBY’ at 120 DAF. Therefore, we focused on the DEGs between the two varieties at this stage. Venn analysis revealed that 1,531 genes, which show differential expression specifically at 120 DAF, have been designated as 120 DAF-preferential (**Figure 3G**). To identify the key regulatory pathways involved in longan sucrose accumulation, we conducted KEGG pathway analysis on the 120 DAF stage-preferential genes. The results indicate that 11 annotated unigenes related to sucrose metabolism (starch and sucrose metabolism) are involved, including genes encoding pectinesterase, beta-glucosidase, endo-(1, 4)-beta-D-glucanase, 1, 4-alpha-glucan-branching, nudix hydrolase, sucrose-phosphate synthase, galacturonosyltransferase, phosphoglucomutase, and UDP-glucuronic acid decarboxylase (**Supplementary Figure S2** and **Supplementary Table S4**). Additionally, the glucose content showed significant differences between ‘SFB’ and ‘QKBY’ during fruit development. A total of 571 common DEGs were identified (**Figure 3H**). KEGG pathway analysis revealed that 5 annotated unigenes related to glucose metabolism (Glucosinolate biosynthesis and Glycolysis/Gluconeogenesis) are involved, including genes encoding UDP-glycosyltransferase, alcohol dehydrogenase, ATP-dependent 6-phosphofructokinase, pyruvate kinase, and hexokinase (**Supplementary Figure S3** and **Supplementary Table S5**).

Moreover, the fructose content in ‘SFB’ and ‘QKBY’ fruits exhibits significant variations at 60 DAF and 120 DAF. Notably, 718 common DEGs were identified in these two stages. KEGG pathway analysis of these genes led to the identification of two genes involved in fructose metabolism (Fructose and mannose metabolism), including one encoding ATP-dependent 6-phosphofructokinase and another encoding hexokinase (**Supplementary Figure S4** and **Supplementary Table S6**).

### 3.5 Combined DEG and WGCNA to confirm the key genes involved in sugar accumulation

**Figure 5:**
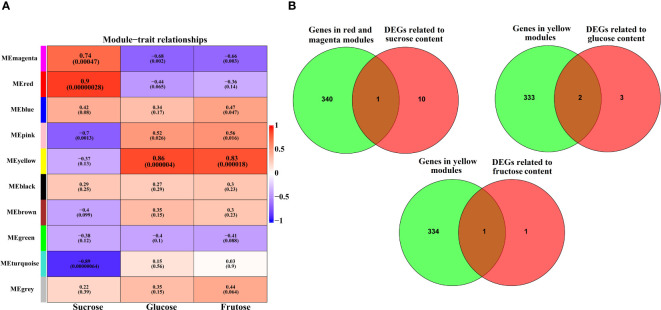
Identification of key genes involved in sugar accumulation. **(A)** C The heatmap of module−sugar relationships. Each row represents a module indicated by different colors. The color key from blue to red represents correlation values from−1 to 1. **(B)** Venn diagram of the key genes in each module with DEGs related to sugar metabolism.

We conducted WGCNA to identify the co-expressed gene modules significantly associated with the content of sucrose, glucose, and fructose. The result identified 10 distinct modules, with the number of genes per module ranging from 134 (magenta) to 4,793 (turquoise) (**Supplementary Figure S5** and **Supplementary Table S7**). Notably, the red (r=0.9, p=0.00000028) and magenta (r=0.74, p=0.00047) module was significantly positively correlated with sucrose content. The yellow module was significantly positively correlated with glucose (r=0.86, p=0.000004) and fructose (r=0.83, p=0.000018) content (**Figure 5A**). To further explore key genes involved in sugar accumulation, venn plot was used to reveal the overlapped filtered genes of significantly modules genes and DEGs. As shown in **Figure 5B**, one gene (*DlSPS*) was identified to be involved in sucrose content. Furthermore, one gene (*DlHK*) and two genes (*DlADH* and *DlHK*) were involved in glucose and fructose content, respectively (**Supplementary Figure S6**).

### 3.6 Transient overexpression of the *DlSPS* gene enhances sucrose accumulation in strawberry fruit

**Figure 6:**
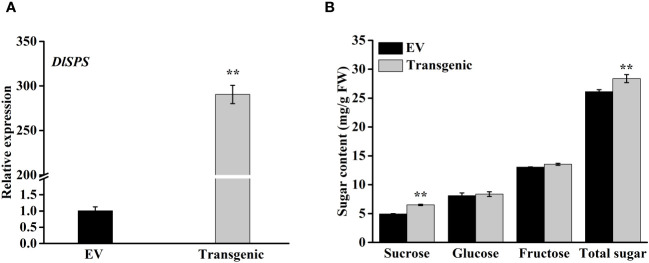
Overexpression of *DlSPS* in strawberry fruit. **(A)** Relative expression of *DlSPS* in strawberry fruits infiltrated with *DlSPS* and the entry vector, respectively. **(B)** Sugar content in control and transgenic strawberry fruits, respectively. Small star above the bars indicate significant differences (** *p* ≤ 0.01).

To examine the possible role of *DlSPS* in fruit sucrose accumulation, we transiently transferred *pSAK277-DlSPS* vector into immature white fruit of strawberry with empty *pSAK277* vector as control. The qRT-PCR analysis showed that *DlSPS* was significantly higher expressed in transgenic fruit than that in the control (**Figure 6A**). The concentrations of sucrose and total sugar in the *DlSPS*-overexpression were significantly higher than those in the control. However, the glucose and fructose showed no significant difference between fruits overexpressing *DlSPS* or empty vector (**Figure 6B**).

### 3.7 Identification of transcription factors involved in sucrose accumulation in longan

**Figure 7:**
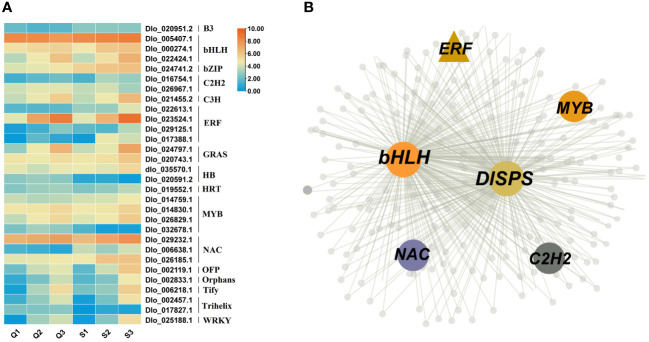
Identification of transcription factors Involved in soluble sugars accumulation. **(A)** Expression profiles of 30 transcription factors in magenta, red and yellow modules. Q1: QKBY at 60 DAF; Q2: QKBY at 90 DAF; Q3: QKBY at 120 DAF; S1: SFB at 60 DAF; S2: SFB at 90 DAF; S3: SFB at 120 DAF. **(B)** The co-expression network contained *DlSPS* genes for the magenta module.

To identify the key transcription factors regulating sugar content in longan, we first identified the transcription factors present in the magenta, red and yellow modules. The results revealed that a total of 30 transcription factors exist in these three modules, including bHLH (3), bZIP (1), ERF (4), MYB (4), NAC (3), and WRKY (1) (**Figure 7A**). As shown in **Figure 5A**, *DlSPS* is key gene regulating sucrose synthesis in longan fruit. Therefore, we investigated the potential regulation pathway between TFs and *DlSPS*. Notably, five TF genes that were categorized in the magenta module (**Figure 7B**) had similar expression trends to *DlSPS*. Furthermore, many binding sites of this five TFs on the promoters of *DlSPS* genes were also identified (**Supplementary Table S8**).

## 4 Discussion

### 4.1 Sugar compositions in the longan pulp among two cultivars

Sweetness, which depends on the soluble sugar components and content, plays crucial role in determining fruit flavor and quality in longan. Various components of soluble sugar have been observed in plants, such as sucrose, fructose, glucose, raffinose, and galactose (Kotha et al., 2020; Zheng et al., 2022). In this study, we found that the main soluble sugars in longan fruit were sucrose, glucose, and fructose, which consistent with previous reports in other fruits such as apples (Ma et al., 2015) and peaches (Vimolmangkang et al., 2016). However, each soluble sugar component showed a distinct accumulation pattern, for instance, the sucrose content increased markedly during fruit development and exhibited significant differences between ‘SFB’ and ‘QKBY’ at the mature stage. While, the sucrose content was notably lower than previously research which conducted on the same two varieties in Guangdong province (Luo et al., 2019). This discrepancy may be caused by different weather and cultivation conditions, such as rainfall, light exposure, temperature, and fertilization. Furthermore, we observed a slight increase in glucose and fructose contents from 60 DAF to 90 DAF, followed by a slight decrease from 90 DAF to 120 DAF. At the mature stage, the contents of glucose and fructose were significantly lower than those of sucrose, consistent with previous research demonstrating that sucrose is the primary component of soluble sugar in longan.

### 4.2 Genes related to soluble sugar metabolism

The sugar accumulation during fruit development is regulated by a variety of biological processes, such as sugar metabolism and transportation (Chen et al., 2021). In previous studies, many important enzymes and transporters have been identified in longan, such as soluble acid invertases (SAI), vacuolar glucose transporters family (VGTs), and sugars will eventually be exported transporters (SWEET) (Luo et al., 2019; Fang et al., 2020a, 2022).

To gain a deeper understanding of the molecular mechanisms of sugar accumulation in longan, a comparative transcriptomic analysis was conducted and identified several potential genes involved in sugar metabolism, including SPS and HK. SPS is a key enzyme in sucrose metabolism. In melon, the activity of SPS is positively correlated with sucrose accumulation (Gao et al., 2024; Wang et al., 2023). In sugarcane, overexpression the *SoSPS1* gene increased sucrose content in leaves (Anur et al., 2020). In this study, we found expression level of one SPS gene and sucrose content were significant differences between two cultivars at 120 DAF, suggesting that the SPS gene may be involved in sucrose metabolism in longan fruit. Furthermore, transient overexpression of the *DlSPS* gene enhances sucrose and total sugar content in strawberry fruit, demonstrating *DlSPS* gene is a strong candidate for the regulation of sugar accumulation in longan fruit. Hexokinase (HK), a multifunctional protein that serves as a sugar sensor for glucose signaling and a catalyst for glycolysis, has also been implicated in sugar metabolism. Overexpression of the *PbHXK1* gene in tomato significantly reduced sugar content in leaves (Zhao et al., 2019). In this study, one HK gene showed significant expression levels between two cultivars during fruit development, indicating its potential function in longan soluble sugar metabolism.

### 4.3 TFs related to sucrose metabolism

Several studies have demonstrated that transcription factors play crucial roles in fruit sugar accumulation. For instance, in apple, *MdbHLH3* modulates the soluble sugar content by activating phosphofructokinase gene expression (Yu et al., 2022), while *MdMYB305* interacts with sugar-related genes, including *MdCWI1*, *MdVGT3*, and *MdTMT2*, thereby enhancing their activities and ultimately increasing the sugar content in fruits (Zhang et al., 2023). *PuWRKY31* bound to the *PuSWEET15* promoter and induced its transcription to increase sucrose content in pear (Li et al., 2020). In present study, three modules, including 30 transcription factors (TFs), were significantly positively correlated with soluble sugar content. Our RNA-seq results revealed that *DlSPS* expression was significantly different between the ‘SFB’ and ‘QKBY’ fruits (**Supplementary Table S4**). The transient overexpression of the *DlSPS* gene in strawberry fruit validated its positive role in regulating sucrose accumulation. Previous studies revealed various TFs participated in sucrose synthesis via binding to promoters of SPS to alter its expression level. For instance, CdWRKY2 is a positive regulator in cold stress by targeting *CdSPS1* promoters and activating its expression to mediate sucrose biosynthesis in bermudagrass (Huang et al., 2022). CmMYB44 repressed the transcriptional activation of *CmSPS1*, leading to lower sucrose content in melon (Gao et al., 2024). In present study, we found that five TFs exist in the same module as *DlSPS* gene and further analysis indicated thatseveral binding sites of this five TFs were found on the promoters of *DlSPS* genes, suggesting that these TFs might be involved in regulating sucrose pathways via interaction with *DlSPS* genes, thus affecting sucrose accumulation in longan. Taken together, these results partially elucidate the sucrose accumulation regulation process in longan and provide a theoretical foundation for fruit quality improvement. Obviously, more in-depth works needed to be carried out to identify our hypothesis.

## Data availability statement

The datasets presented in this study can be found in online repositories. The names of the repository/repositories and accession number(s) can be found in the article/**Supplementary Material**.

## Author contributions

YL: Writing – original draft, Investigation, Methodology. RR: Investigation, Methodology, Writing – original draft. RP: Investigation, Writing – original draft. YB: Investigation, Writing – original draft. TX: Investigation, Writing – original draft. LZ: Writing – original draft, Project administration, Writing – review & editing. TF: Project administration, Writing – original draft, Writing – review & editing.
