# Supplementary material for: Comparative transcriptome analysis identifies candidate genes related to sucrose accumulation in longan (Dimocarpus longan Lour.) pulp
Source: Front Plant Sci. 2024 Apr 5;15:1379750. doi: 10.3389/fpls.2024.1379750 (PMC11032017; doi:10.3389/fpls.2024.1379750)
Supplement: Supplementary file 1 [file DataSheet_1.docx]

**Table. S1. The primers used in this study.**

| **Primer** | **Forward primer (5’-3’)** | **Reverse Primer (5’-3’)** |
| --- | --- | --- |
| DlbHLH68 | CGCAGCAAACAGCTTCGTAT | AGGATGCCTCCCATCTGACT |
| DlbHLH63 | AATGAAAGGGTGTGCAGAGGA | TGAACCTCAGAAACCTTGGAGT |
| DlNAC73 | ATGGTCAAATCCGCCACTTCT | ACCCTTGACAGTTCCATCACC |
| DlVGT1 | TGCTTGTGAATTTTGGCGCA | TTGTCTCCGGAACGATGACG |
| DlPLT5 | TCTCGGAGCAGTGGGAAGTA | AAGCGATGGAAAGAGACGCA |
| DlSTP1 | AGGCTTCTCAGTGTTGGTGG | TGATGCTTTGTCCGGCTGAT |
| DlSWEET1 | TACTGGCGGTGTTTTCTGGG | AGAGAGGGGTGAGGCATACA |
| DlSPS1 | AACTCATTCCTCGTCTCGGG | ACTGAAGCTACCCAACGAATCA |
| DlSUT2 | CCGTGATAAGTTCCCGCCTT | CAAGACCCGTGCTATTGGGT |
| DlTMT1 | AAGCCAGCCCGCTCTTTATT | GGATCAAGAAGAGCAGCCCA |
| Actin | TGCTATCCTTCGGTTGGACC | CGGACGATTTCCCGTTCAG |
| DlSPS-pSAK277 | TCCAAAGAATTCAAAAAGCTTAT  GGCAGGAAATGACTGGATAAA | TCATTAAAGCAGGACTCTAGACTAGG  CCTTAAGAATTCCTAAACTCTC |
| DlSPS-qRT-PCR | AACTCATTCCTCGTCTCGGG | ACTGAAGCTACCCAACGAATCA |
| Actin- strawberry | ACCGTTGATTCGCACAATTGGTCATCG | TACTGCGGGTGGCAATCGGACG |

**Table. S2. Summary statistics of RNA-Seq results**

| **Stage** | **Group name** | **Samples** | **Clean reads** | **Mapped Reads** | **GC Content** | **%≥Q30** |
| --- | --- | --- | --- | --- | --- | --- |
| 60 DAF | Q1 | Q1-1 | 22462364 | 40,606,187 | 0.4567 | 0.9513 |
|  |  | Q1-2 | 20418767 | 36,778,266 | 0.4503 | 0.9509 |
|  |  | Q1-3 | 21265359 | 38,629,556 | 0.4517 | 0.9474 |
| 90 DAF | Q2 | Q2-1 | 22859363 | 41,976,340 | 0.4514 | 0.9522 |
|  |  | Q2-2 | 20836671 | 38,421,651 | 0.454 | 0.9501 |
|  |  | Q2-3 | 22106133 | 40,800,283 | 0.4502 | 0.9507 |
| 120 DAF | Q3 | Q3-1 | 60373333 | 90,336,229 | 0.4449 | 0.9557 |
|  |  | Q3-2 | 61615865 | 89,432,656 | 0.4454 | 0.9532 |
|  |  | Q3-3 | 58644215 | 95,552,783 | 0.4456 | 0.9553 |
| 60 DAF | S1 | S1-1 | 23299996 | 42,663,126 | 0.4614 | 0.9469 |
|  |  | S1-2 | 24455909 | 44,300,137 | 0.4535 | 0.946 |
|  |  | S1-3 | 22457531 | 40,542,581 | 0.4536 | 0.946 |
| 90 DAF | S2 | S2-1 | 22575456 | 41,044,795 | 0.4503 | 0.948 |
|  |  | S2-2 | 21886361 | 40,257,309 | 0.4498 | 0.9475 |
|  |  | S2-3 | 23774467 | 43,805,157 | 0.4494 | 0.9502 |
| 120 DAF | S3 | S3-1 | 77037023 | 131,468,938 | 0.4597 | 0.9563 |
|  |  | S3-2 | 64338406 | 107,228,196 | 0.4419 | 0.948 |
|  |  | S3-3 | 81405449 | 130,473,621 | 0.4433 | 0.9547 |

**Table. S3. Statistics of new gene function annotation**

| **Annotated databases** | **New Gene Number** |
| --- | --- |
| NR | 2,539 |
| eggNOG | 2,204 |
| Swiss-Prot | 1,465 |
| KOG | 1,448 |
| GO | 1,369 |
| Pfam | 1,216 |
| KEGG | 669 |
| COG | 363 |

**Table. S4. Annotation of 11 unigenes related to starch and sucrose metabolism**

| Gene ID | Annotation |
| --- | --- |
| Dlo_002752.1 | endo-(1,4)-beta-D-glucanase |
| Dlo_004429.1 | 1,4-alpha-glucan-branching |
| Dlo_005576.1 | nudix hydrolase |
| Dlo_006581.1 | beta-glucosidase |
| Dlo_018817.1 | pectinesterase |
| Dlo_019085.1 | sucrose-phosphate synthase |
| Dlo_020799.1 | galacturonosyltransferase |
| Dlo_023353.1 | pectinesterase |
| Dlo_026011.1 | beta-glucosidase |
| Dlo_028830.1 | phosphoglucomutase |
| Dlo_033392.1 | UDP-glucuronic acid decarboxylase |

**Table. S5. Annotation of 5 unigenes related to glucosinolate biosynthesis and glycolysis/gluconeogenesis**

| Gene ID | Annotation |
| --- | --- |
| Dlo_000737.1 | UDP-glycosyltransferase |
| Dlo_020097.3 | alcohol dehydrogenase |
| Dlo_025902.2 | ATP-dependent 6-phosphofructokinase |
| Dlo_033042.1 | pyruvate kinase |
| dlo_035234.1 | hexokinase |

**Table. S6. Annotation of 2 unigenes related to fructose and mannose metabolism**

| Gene ID | Annotation |
| --- | --- |
| Dlo_025902.2 | ATP-dependent 6-phosphofructokinase |
| dlo_035234.1 | hexokinase |

**Table. S7. The gene numbers in each module**

| Module | Gene number |
| --- | --- |
| Black | 186 |
| Blue | 1515 |
| Brown | 1078 |
| Green | 212 |
| Grey | 1400 |
| Magenta | 134 |
| Pink | 140 |
| Red | 207 |
| Turquoise | 4793 |
| Yellow | 135 |

**Table. S8. The statistics of transcription factor binding sites in the promoter of *DlSPS***

| Transcription factor | Binding site | Number |
| --- | --- | --- |
| bHLH | CACCTG | 2 |
| NAC | CGT/(G/A) | 6 |
| MYB | CAACAG | 1 |
| ERF | TGTTG | 3 |
| C2H2 | CTCAGTCT | 1 |

**
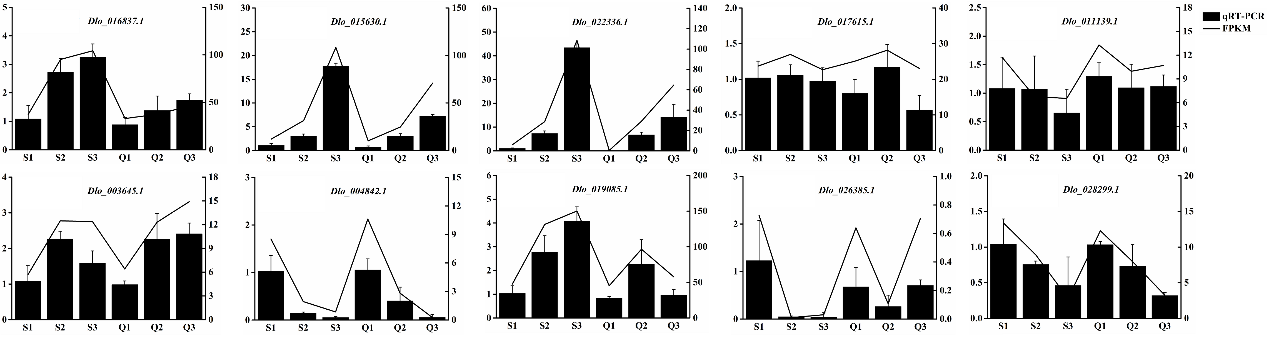
**

**Figure. S1. Verification of transcriptomic data by qRT-PCR analysis of 10 gene expression.**

**
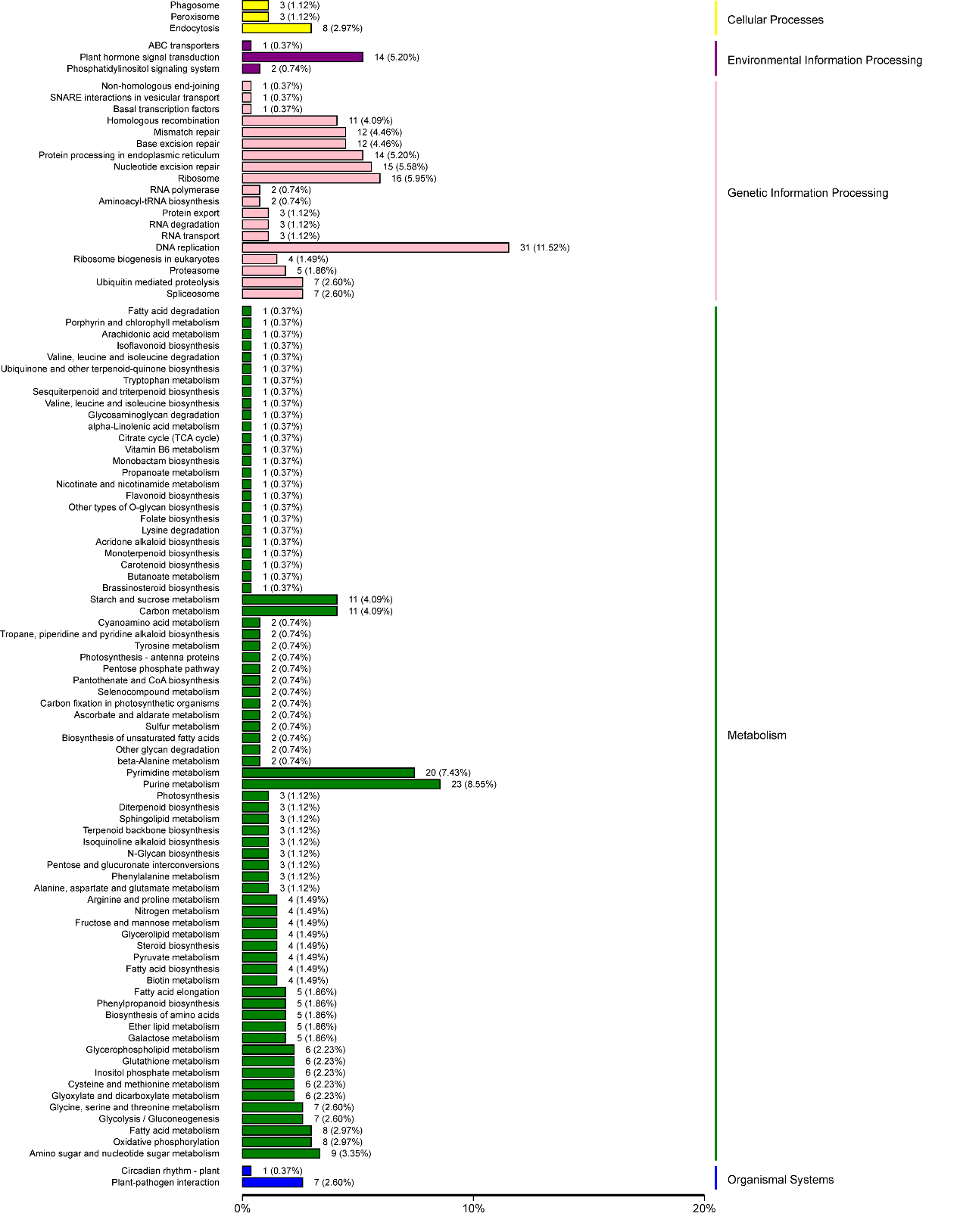
**

**Figure. S2. The KEGG class of 1,531 genes, which show differential expression specifically at 120 DAF. The numerals beside the histogram indicate the number of DEGs.**

**
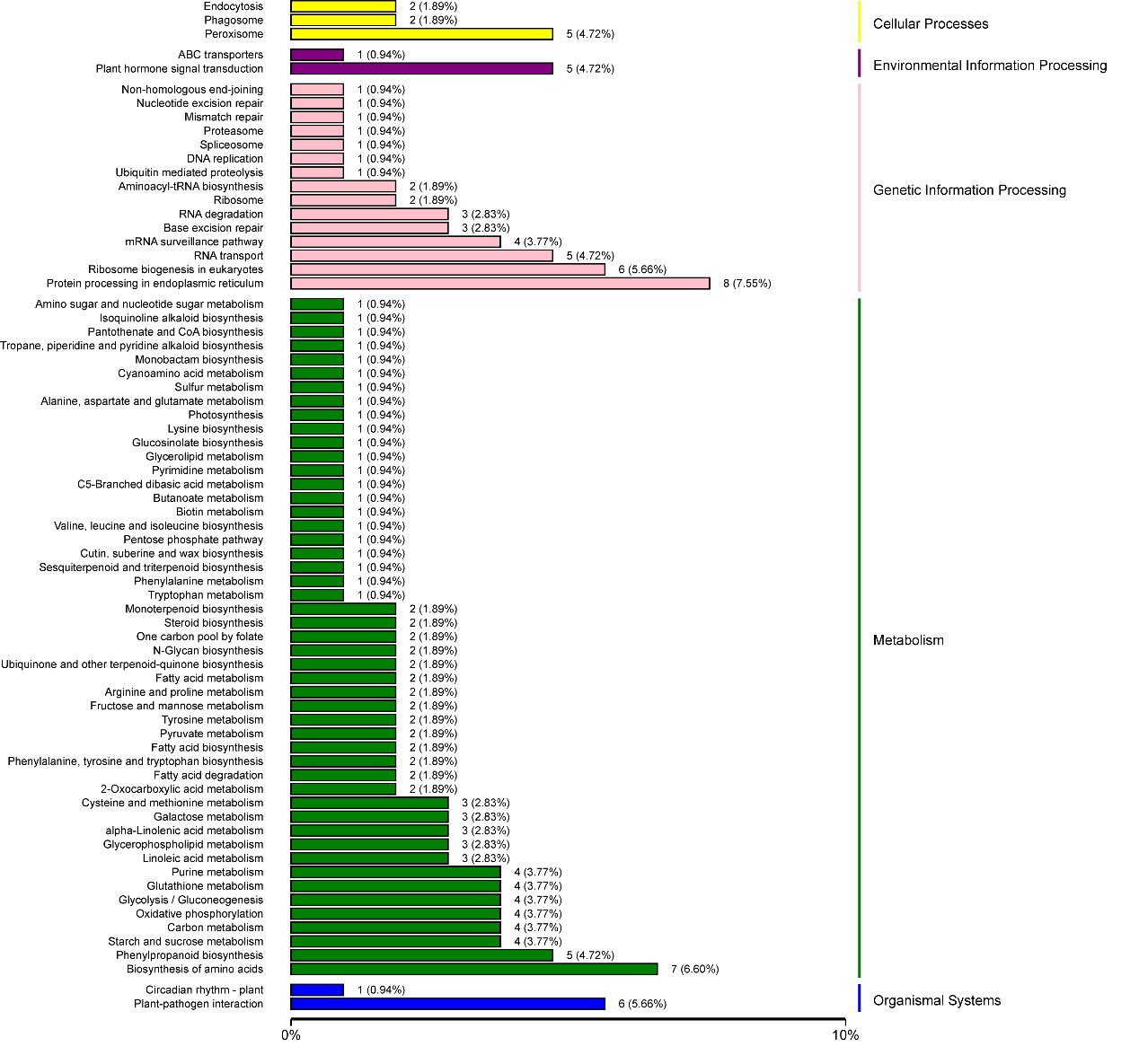
**

**Figure. S3. The KEGG class of 571 common DEGs** **between 'SFB' and 'QKBY' during fruit development. The numerals beside the histogram indicate the number of DEGs.**

**
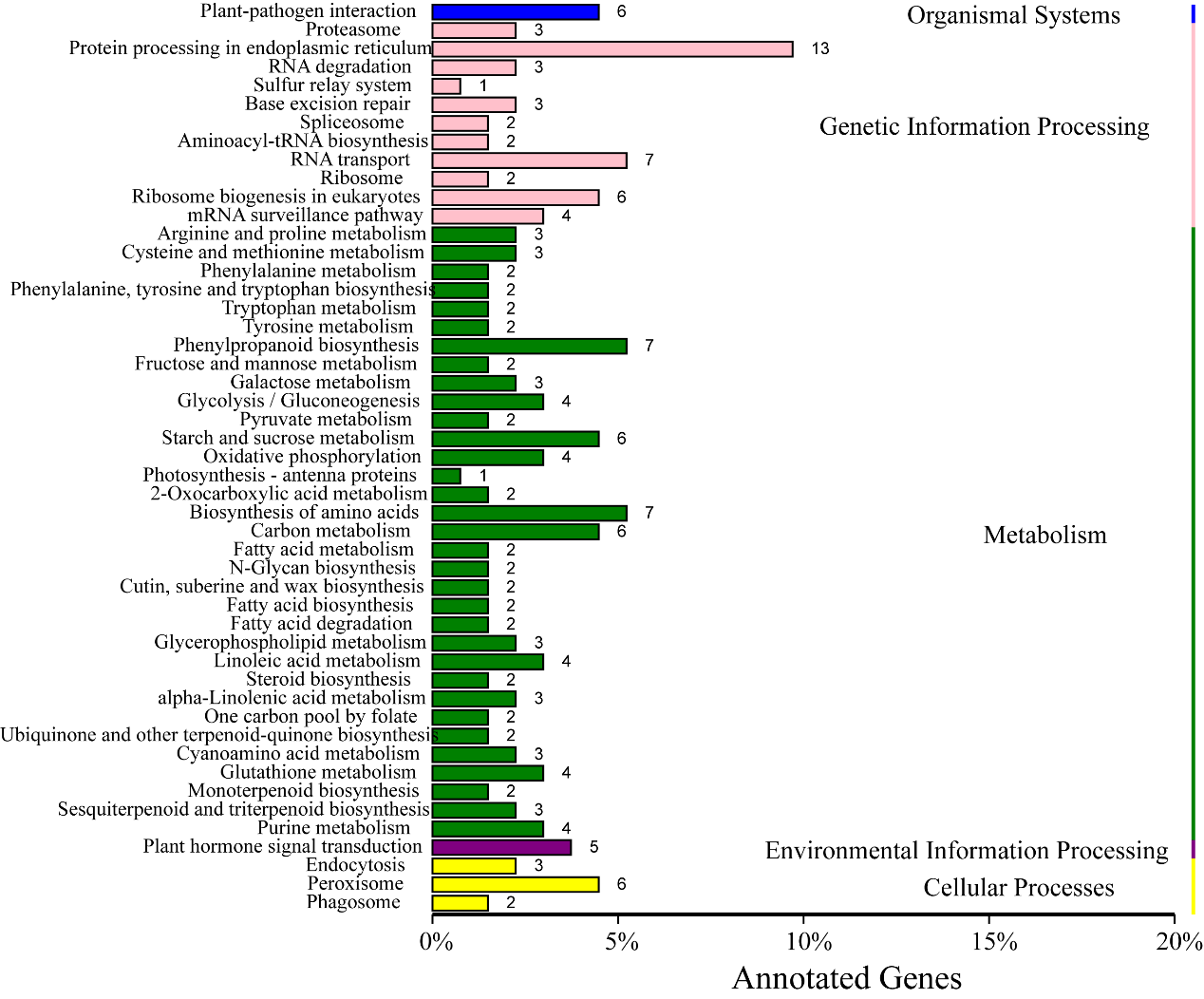
**

**Figure. S4. The KEGG class of 718 common DEGs** **between 'SFB' and 'QKBY' at 60 DAF and 120 DAF. The numerals beside the histogram indicate the number of DEGs.**

**
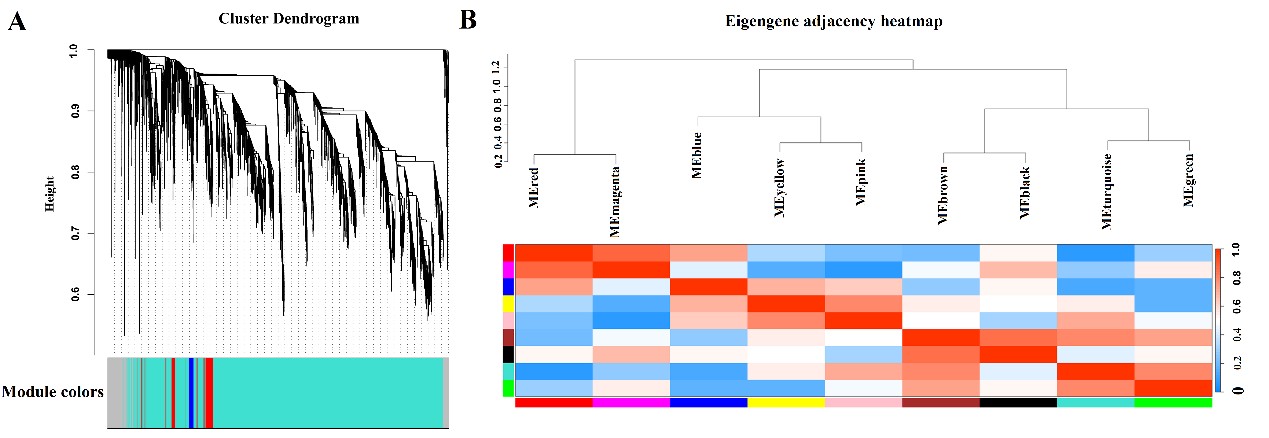
**

**Figure. S5. WGCNA of the gene expression matrix.** (A) Hierarchical clustering tree dendrogram) illustrating 10 modules of co-expressed genes. (B) Clustering tree analysis of different modules.


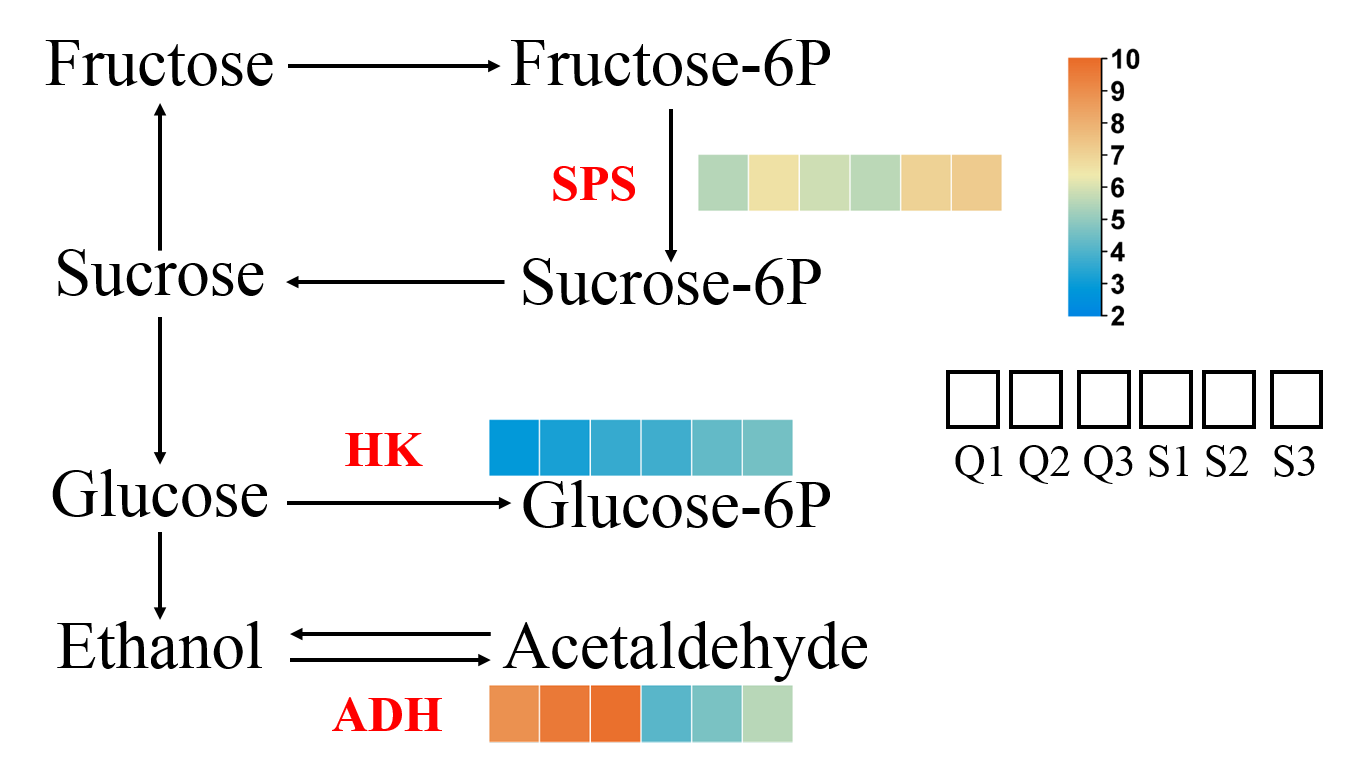


**Figure. S6. The key genes involved in sugar metabolism pathways.** The heatmaps represent the expression of key genes in two cultivars at three developmental stages,respectively. Q1: QKBY at 60 DAF; Q2: QKBY at 90 DAF; Q3: QKBY at 120 DAF; S1: SFB at 60 DAF; S2: SFB at 90 DAF; S3: SFB at 120 DAF.
